# Maternal genetic and phylogenetic characteristics of domesticated cattle in northwestern China

**DOI:** 10.1371/journal.pone.0209645

**Published:** 2018-12-27

**Authors:** Yuan Cai, Ting Jiao, Zhaomin Lei, Li Liu, Shengguo Zhao

**Affiliations:** 1 College of Animal Science & Technology, Gansu Agricultural University, Lanzhou, Gansu, PR China; 2 College of Grassland, Gansu Agricultural University, Lanzhou, Gansu, PR China; National Cheng Kung University, TAIWAN

## Abstract

Northwestern China, an important part of the Silk Road, was the birthplace of the Zhouzu farming culture. The domestication of cattle as an important aspect of farming culture has had a long history in northwestern China. In this study, we assessed the maternal structure and phylogeny of cattle by analyzing the mitochondrial DNA hypervariable segment I (HVS-I) in 698 native cattle from eight areas of northwestern China. The phylogenetic analyses revealed two highly divergent mtDNA clades: clade T, which had four sub-clades (Ta—Td), and clade I. The cattle domesticated from *Bos taurus* showed a clear dominant distribution pattern in northwestern China. The nucleotide diversity of the *Bos indicus* clade was lower than that of clades from *Bos taurus*. In summary, our results suggest that the native cattle of northwestern China were domesticated from two different maternal ancestors, *Bos taurus* and *Bos indicus*, which migrated to the central plains of China from the north and south, respectively, with *Bos taurus* remaining at the edges of the region. The population expansion of the cattle domesticated from *Bos taurus* occurred in the Longdong region of Gansu Province, and these cattle formed four relatively independent evolutionary branches. Subsequent to this expansion event, *Bos indicus* migrated from southern to northern China.

## Introduction

The breeding of animal genetic resources (AnGR) for human use began approximately 12 000 to 14 000 years ago during the agricultural revolution of the early Neolithic age with the domestication of major crop and livestock species [1]. The domestication of animals and plants is considered one of the most important developments in history and one of the prerequisites for the rise of human civilizations [2]. After the initial domestication events, farming rapidly spread into nearly all terrestrial habitats [3]. Thousands of years of natural and human selection, genetic drift, inbreeding and cross-breeding have contributed to AnGR diversity and have allowed livestock to be cultivated in a variety of environments and production systems. The Chinese native cattle represent an essential element of early agricultural production systems and were especially important in the development of early farming [4]. A previous study showed that taurine cattle arrived in China aproximately 5000 years ago [5], and a bovine jaw dated 10 500 BP found in Northeast China showed clear signs of the stereotypical bar biting often displayed by captive animals and contained taurine mtDNA from a hitherto unknown mtDNA haplogroup, suggesting independent and early domestication [6]. However, recently, the East Asian cattle populations were discovered to be mainly composed of three distinct ancestries, including an earlier East Asian taurine ancestry that reached China at least ~3.9 kya, a later introduced Eurasian taurine ancestry, and a novel Chinese indicine ancestry that diverged from the Indian indicine approxi-mately 36.6–49.6 kya [7]. Cattle as a draught animal and tool of production were domesticated and reared in northwestern China. Although, hybridization was performed with exotic breeds (sire or semen) in the past two decades, this case did not affect the maternal genetic relationships. Thus, the domestication and migration of cattle are closely linked to farming culture, and a systematic study of the maternal genetic and phylogenetic characteristics of domesticated cattle was conducted to understand the history of cattle breeds and populations, especially in northwest China.

## Materials and methods

### Sampling

All animal work was conducted according to the guidelines for the care and use of experimental animals established by the Ministry of Science and Technology of the People’s Republic of China (Approval number 2006–398) and was approved by the Animal Care Committee of Gansu Agricultural University.

Blood samples were collected from 525 cattle distributed in 10 indigenous populations according to their body characteristics, and the data from these samples were combined with the pedigree records provided by producer households and breeding enterprises in the Loess Plateau of western China, including the Gansu, Shanxi, Ningxia and Qinghai Provinces (S1 Fig, S1 Table). Additionally, 173 mtDNA D-loop sequences published in GenBank (19 mtDNA sequences from Gansu native cattle, 18 mtDNA sequences from Inner Mongolia native cattle, one mtDNA sequence from Qinghai native cattle, 19 mtDNA sequences from Shanxi native cattle, 97 mtDNA sequences from Sichuan native cattle and 19 mtDNA sequences from Xinjiang native cattle) were analyzed together with the other data to enhance reliability.

### Sequencing of the mtDNA D-loop region

Total DNA was extracted from blood samples using the standard phenol chloroform method as described by the Molecular Cloning Laboratory Manual [8]. The mtDNA D-loop hypervariable segment (HVS) sequence was amplified and sequenced using the primers L15738 (Forward 5’- CTGCAGTCTCACCATCAACC-3’) and H493 (Reverse 5’- GTGTAGATGCTTGCATGTAAGT-3’) [9], The numbers in the primer names indicate the positions of the 3’ ends of the primers in the mtDNA complete sequences [10]. The PCR mixture (50 μL) contained 22 μL of ddH2O, 1 μL of forward primer (10 pmol/μL), 1 μL of reverse primer (10 pmol/μL), 25 μL of premixed polymerase (Takara, Dalian, Liaoning, PRC) and 1 μL of DNA (50 ng/μL). The PCR reaction procedure consisted of a denaturation step at 94 °C for 3 min, 35 cycles of 30 s at 94 °C, 1 min at 51 °C for annealing, and 1 min at 72 °C, and an elongation step of 10 min at 72 °C in the last cycle. Then, the products were stored at 4 °C. The PCR products were detected with 2% agarose gel electrophoresis and were then purified and sequenced (Sangon Biological Engineering, Inc. Shanghai, PRC).

### Data analysis

The raw sequences obtained from the ABI3130xl Genetic Analyzer were edited and aligned using the Chromas version 2.51 (http://www.technelysium.com.au/chromas.html) and ClustalX version 2.0 programs [11], respectively. A total of 525 sequence were submitted to GenBank (Genbank accessions MH922224—MH922748). A neighbor-joining (NJ) tree of all haplotypes was constructed with the roots of *Bison bonasus* (AF083356, European bison), *Bos gaurus* (AF083371, gaur) and *Bos grunniens* (AF083355, domestic yak) under the Kimura 2-parameter model (bootstrap = 1000) using MEGA 7.0 [12]. Then, the median-joining networks were constructed using the program Network 5.0 [13] to probe the possible relationships among the major clades. The nucleotide diversity (π) was calculated for the major clades (N>30) using Arlequin 3.5.2.2 [14]. Finally, to detect signatures of population expansion, Fu’s Fs test [15] was also applied using Arlequin 3.5.2.2.

## Results

Total of 525 mtDNA HVS sequences of native cattle from Gansu, Ningxia, Qinghai and Shanxi Provinces were obtained (Table 1), and 121 variable sites were detected. These insertion/deletions (indels) were discarded in the subsequent analyses. A combination of our data and published data yielded 130 variable sites and 258 haplotypes from a total of 698 indigenous cattle (S2 Table).

**Table 1:** Geographical distribution of the major clades in native cattle of northwest China.

Clade			Xinjiang (N = 19)	Hexi of Gansu^a^ (N = 15)	Inner Mongolia (N = 18)	Ningxia (N = 75)	Qinghai (N = 86)	Longdong of Gansu^b^ (N = 211)	Shanxi (N = 177)	Sichuan (N = 97)	Total No. (N = 698)
T	Ta	Individual^c^ (%)	13 (68.42)	6 (40.00)	9 (50.00)	31 (41.33)	25 (29.07)	77(36.49)	60 (33.90)	20 (20.62)	241
		Haplotype^d^	4 (3)	3 (1)	6 (0)	21 (13)	21 (10)	48 (27)	40 (23)	11 (4)	
	Tb	Individual (%)	2 (10.53)	3 (20.00)	4 (22.22)	6 (8.00)	11(12.79)	31(14.69)	13 (7.34)	4 (4.12)	74
		Haplotype	1 (1)	3 (1)	3 (0)	5(2)	9 (4)	22 (9)	11 (3)	2 (1)	
	Tc	Individual (%)	1 (5.26)	5 (33.33)	4 (22.22)	8 (10.67)	18 (20.93)	31 (14.69)	30 (16.95)	32 (32.99)	129
		Haplotype	1 (0)	5 (2)	1 (1)	7 (6)	9 (5)	16 (11)	14 (11)	14 (8)	
	Td	Individual (%)	1 (5.26)	1 (6.67)	0	14 (18.67)	12 (13.95)	27 (12.80)	18 (10.17)	11 (11.34)	84
		Haplotype	1 (0)	1 (0)	0	11(9)	6 (3)	14 (11)	7 (4)	4 (2)	
	Total	Individual (%)	17 (89.47)	15 (100)	17 (94.44)	59 (78.67)	66 (76.74)	166 (78.67)	121 (68.36)	67 (69.07)	528
		Haplotype	7 (4)	12 (4)	10 (1)	44 (30)	45 (22)	99 (58)	72 (41)	31 (15)	
I		Individual (%)	2 (10.53)	0	1 (5.56)	16 (21.33)	20 (23.26)	45 (21.33)	56 (31.64)	30 (30.93)	170
		Haplotype	1 (0)	0	1 (1)	5 (1)	7(3)	14 (6)	15 (9)	6 (2)	

*Note*. The number of haplotypes and unique haplotypes for the major phylogenetic clades in the different regions were counted based on S1 and S2 Tables. Haplotypes are defined by substitutions only, disregarding indels.

^a^ Hexi of Gansu include Anxi and Wuwei.

^b^ Longdong of Gansu includes Pingliang and Qingyang.

^c^ Number of samples and its proportion in the clade (in parentheses).

^d^ Number of haplotypes and unique haplotypes (in parentheses)

### Phylogeny and network profiles of the clades

The alignment of *Bos taurus* (Accession No.: V00654 [10]) and *Bos indicus* (Accession No.: L27733 [16]) sequences indicated that 258 haplotypes were in the *Bos taurus* (T) and *Bos indicus* (I) clades. Surprisingly, four sub-clades (Ta, Tb, Tc and Td) were represented by the characteristic variable sites in clade T (S2 Table). Ta, Tb and Tc were found in all eight areas in northwest China. Mongolia cattle were not found in clade Td, and Hexi cattle from Gansu were not found in clade I.

**Fig 1:**
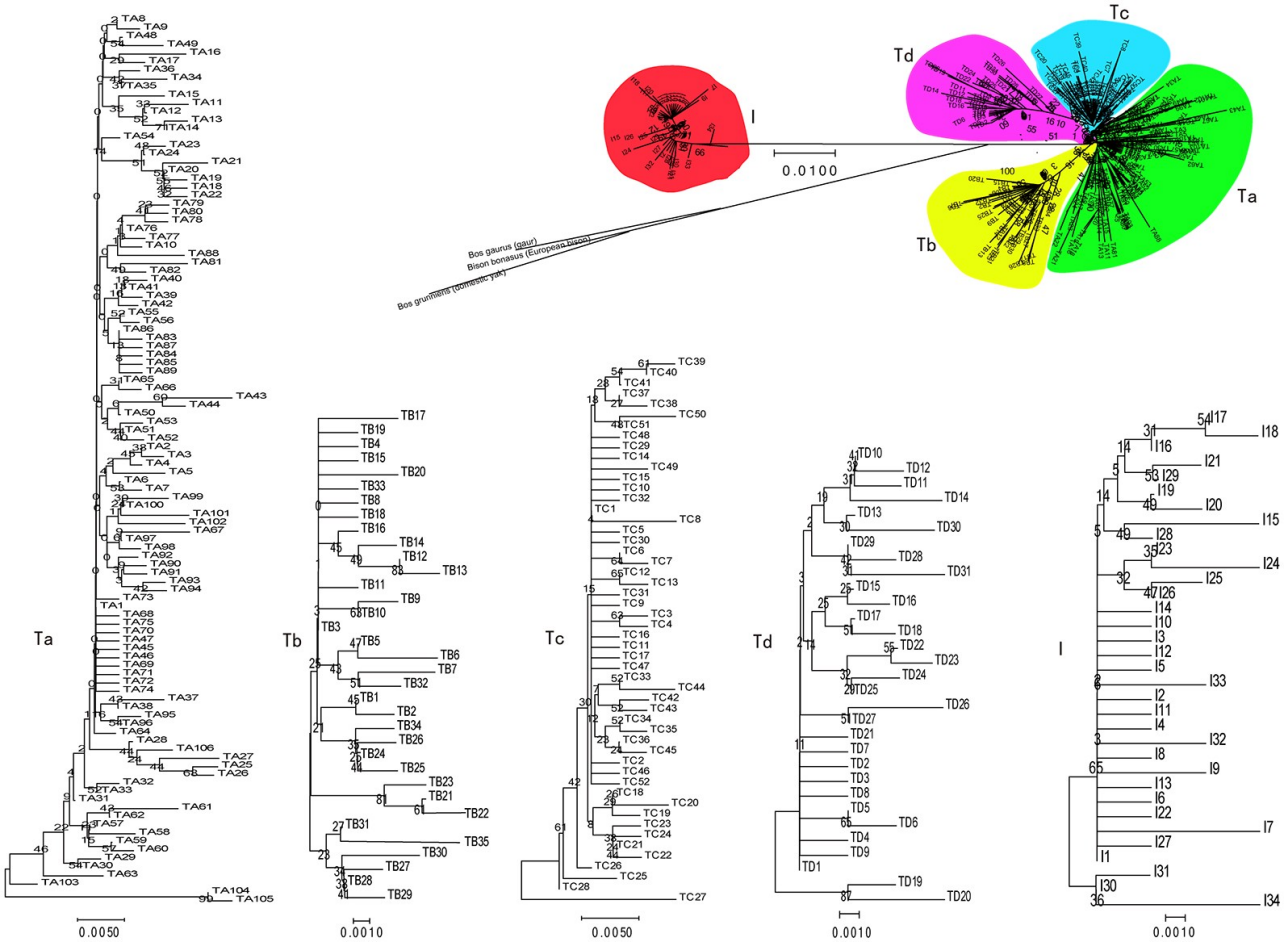
Neighbor-joining (NJ) tree of 258 haplotypes in 698 domestic cattle (*Bos taurus taurus*). The highly divergent mtDNA clades were marked with Ta—Td and I. The neighbor-joining (NJ) tree of all haplotypes was constructed with the roots of *Bison bonasus* (AF083356, European bison), *Bos gaurus* (AF083356, gaur) and *Bos grunniens* (AF083356, domestic yak).

**Fig 2:**
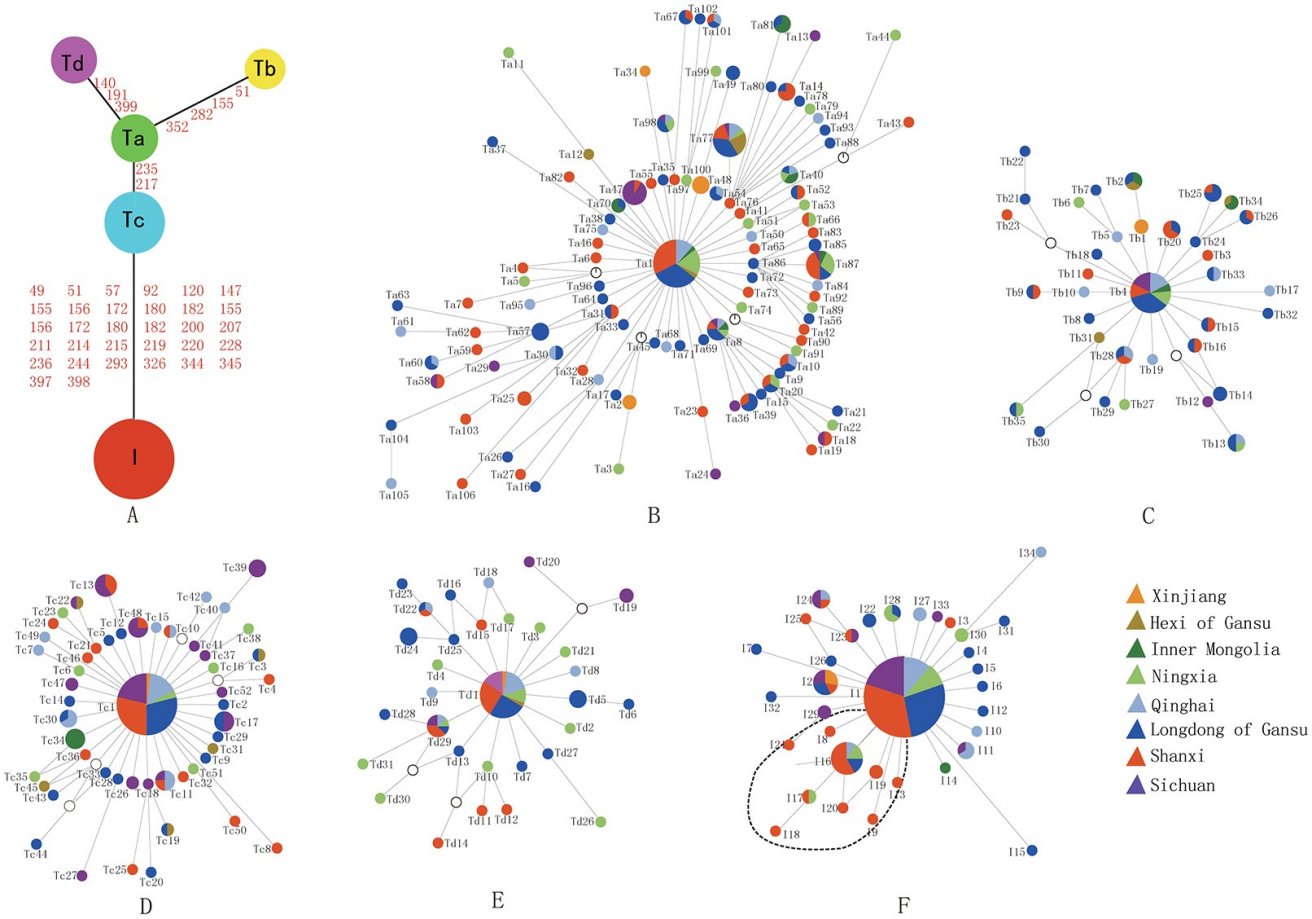
Network profiles of the major clades. The links are labeled by the nucleotide positions to designate transitions; transversions are further specified by adding suffixes A, G, C and T; recurrent mutations are underlined. The order of the mutations on a branch is arbitrary. Circled areas are proportional to haplotype frequencies. (A) Overall schematic profile of the major clades (Ta—Tc and I). Networks of the respective major clades. The locations of the samples are demonstrated by different colors. The mark “¤” refers to the potential root. For interpretation of the references to color in this figure legend, the reader is referred to the web version of this paper.

The NJ tree for the 258 haplotypes (Fig 1) revealed five divergent clades (Ta-Td, I). The analysis showed that the potential roots of each of the five clades differed from each other by at least two mutations (Fig 2A). In each of the five clades (Ta, Tb, Tc, Td and I), a dominant haplotype was observed that had a relatively wider geographic distribution; the number of native cattle sharing that haplotype ranged from 17 to 96 (Fig 2, S1 Table). However, clade Ta was much more variable, and the number of samples that shared a haplotype was less than or equal to 17. With the exception of six samples (3, 2 and 1 samples from Ningxia, Longdong and Qinghai Provinces, respectively), nine haplotypes in clade I were observed in only one sample (Fig 2F).

Clade A showed even larger distances within the clade (S2 Table, Fig 2), and the largest distance between the Tb, Tc and Td sub-clades and the Ta root in clade T was no more than 4 mutations. However, the distance from sub-clade Tc, which was the smallest in clade T, to clade I reached 32 mutations (Fig 2).

### Geographic distribution of the clades

Compared with chickens [17, 18] and Baltic Sea region cattle [19, 20], a regional distribution was observed in the clades of the studied animals, which indicated that geographic structuring occurred in the studied cattle populations. In general, all clades showed a wide geographical distribution. However, clade Ta dominated in Xinjiang (68.42%) and Inner Mongolia (50%). Clade Tb was primarily composed of cattle samples from Hexi, Gansu Province, but also included samples from Inner Mongolia cattle. Clade Tc was chiefly distributed in Sichuan and in Hexi, Gansu Province. Clade Td was found in each region except for Inner Mongolia, and clade I was mainly distributed in Shanxi and in Sichuan but was absent from Hexi, Gansu Province.

As described in Table 1, six of the eight regions (except Inner Mongolia and Hexi, Gansu Province) contained all five clades harboring most of the domestic cattle samples. In the widely distributed clade A, the proportion of unique haplotypes from the Longdong region of Gansu Province was relatively higher than that of the haplotypes from other places. Most of the Xinjiang and Inner Mongolia sequences fell in clade Ta. A considerable number of samples from Shanxi clustered in clades Ta and I and harbored a high proportion of private haplotypes (23/40 in clade Ta and 9/15 in clade I.

### Genetic diversity and expansion test

We estimated the nucleotide diversity for each main clade (Table 2). The results showed that the nucleotide diversities among the clades varied substantially (0.01043–0.02609). Generally, clade I (*Bos indicus* clade) had the lowest nucleotide diversity, and clade Ta (*Bos taurus* clade) had the highest nucleotide diversity. Fu’s Fs test [15] of the five clades harboring the domestic cattle samples was statistically significant (P<0.05) and consistent with their (roughly) star-like network profiles (Fig 2), which suggested that population expansion likely occurred in the past.

**Table 2:** Nucleotide diversity and Fu’s *Fs* test of the major clades in native cattle.

Clade	No.	π^a^	*Fs* ^b^
Ta	241	0.02609±0.01485	-25.85961*
Tb	74	0.01947±0.01176	-26.81826*
Tc	129	0.01407±0.00903	-27.60330*
Td	84	0.01424±0.00916	-27.58500*
I	170	0.01043±0.00719	-28.54692*

^a^ The estimation was restricted to a 411-bp fragment (relative to position 120–530 in the reference sequence (Accession No.: L27733, Loftus et al., 1994. Reference [16]).

^b^ Fu’s *Fs* test (Fu, 1997 Reference [15]).

* Fu’s *Fs* statistic reached significance (P<0.05, and P<0.02 in coalescent simulation).

## Discussion

### Maternal structures of native cattle in northwest China

Based on the domestication of cattle that occurred within Europe and Africa, this study revealed matrilineal lineages from northwest China that may help reveal the maternal structure. Upon comparing the clades (Ta-Td) in our study with the haplogroups (T1-T4) reported in European cattle [7, 20], Tb and Td were dropped into T2 and T4, respectively. Although Ta and Tc were isolated by the two variations, they dropped simultaneously into T3, and T1 was not found in our research. These findings suggested that there is a close relationship between the cattle in northwest China and European cattle. In fact, the four main haplogroups (T1A, T2, T3 (including T3A and T3B) and T5), were found in taurines from six Asian countries (Japan, Korea, Mongolia, Nepal, India and China) according to variations in the mtDNA D-loop[21]. Furthermore, the whole-genome resequencing revealed that the East Asian taurine cattle including three native cattle breeds from northwest China mainly belonged to the T3 (Tc) and T2 (Tb) haplogroups [22].

Our data confirm that the clades of cattle appear to have been replicated in several locations, which is similar to most livestock species, and that these clades occur in several divergent lineages [23–25].

Overall, the analyzed data fit into two main clades (Fig 1): one formed by the *Bos taurus* clade T and another formed exclusively by the *Bos indicus* clade. The mean distances between these two clusters were larger than the distances among the subclades (Ta—Td). Asian cattle breeds were derived from cattle domesticated in the Indian subcontinent or imported from the Fertile Crescent and Europe. Cattle in northern China are primarily of *Bos taurus* ancestry [26], which entered northern China from west Asia [16], and cattle from southern China are predominantly of *Bos indicus* ancestry [26], which entered southern China from the Indian subcontinent [27]. The analysis showed that higher frequencies (more than 80%) of northern China cattle distributed in Xinjiang (89.47%), Hexi (100%) and Inner Mongolia (94.44) were distributed in clade T (Table 1), and lower frequencies from other regions were observed in this clade. These results were opposite from the findings for clade I and suggested that the frequencies of cattle falling into clade T (*Bos taurus* clade) decreased from north to south, whereas the frequencies in clade I (*Bos indicus* clade) decreased from south to north, thus revealing an immigration pattern in which the cattle domesticated in both northern and southern China spread to the south [28]; these phenomena were related to the immigration of humans, the propagation of farming culture, and trade along the Silk Road. The frequencies of cattle from Sichuan, Shanxi, the Longdong region of Gansu and Qinghai, which lie to the north of Central China, that were distributed in clade T were greater than 60% and lower than the frequencies observed in cattle from northern China, because the cited regions represented typical intersection areas of clades T and I and *Bos taurus* × *Bos indicus* hybrids [26]. Similar phenomena were also observed in European cattle [29]. Our data indicate that the cattle in northwest China have maternal *Bos taurus* and *Bos indicus* ancestors, which is consistent with the results for metacentric and telocentric chromosomes [30] confirmed by Lai *et al*. [31].

### mtDNA landscape patterning

Generally modern cattle are accepted to have originated from two domestication events that occurred in the Near East and Africa (*Bos taurus*) and the Indian subcontinent (*Bos indicus*) based on mtDNA [16, 32], microsatellite [33] and chromosome data [30]. Our study also shows that these two strict matrilineal genetic backgrounds are evident in local cattle populations in northwestern China. Surprisingly, four clades (Ta-Td) with close phylogenetic relationships were detected in the *Bos taurus* clade, which indicated that these lineages originated from the same ancestral population.

Along with the estimated expansion events based on the *Fs* test (Table 2) and the roughly star-like network profiles of clades (Fig 2, Table 1), these findings indicate that population expansions occurred in the Longdong region of Gansu Province, where the greatest number of haplotypes and unique haplotypes in each *Bos taurus* clade were observed among the areas in this study. In general, agricultural expansion involved the movement of human populations and cultural exchanges between populations, as illustrated by the adoption of farming by many hunter—gatherer societies [3]. The Longdong region, the main productive area of Zaosheng cattle, represents a confluence of Fuxi culture, Yan Di culture and West Queen culture, and it is also the birthplace of the Zhouzu farming culture. The Bos taurus was immigrated to this region from Northern China with migration and trade among human populations on the Silk Road, and then the expansion event occurred in Longdong. Therefore, cattle, which were used as draught animals, were further domesticated and dispersed to neighboring areas. This process included human migrations and ancient overland trading networks, which played an important role in the dispersion of livestock species. The domestication of livestock enabled large-scale overland trading between civilizations, and livestock were often a traded product [1].

Compared with cattle in other regions, the cattle of the Longdong region of Gansu Province were extensively sampled; however, few haplotypes belonging to clade I were found in cattle from Longdong (21.33), and the frequency of Longdong cattle distributed in clade I was lower than those of cattle from Sichuan, Shanxi and Qinghai (Table 1). Cattle have experienced rapid recent decreases in effective population size from their large ancestral populations because of bottlenecks associated with domestication, selection, and breed formation. Domestication and artificial selection appear to have left signatures of selection, and the current levels of diversity reveal the history of domestication [34]. The low diversity (0.01043) and haplotype number observed in clade I implied that *Bos indicus* originated in the south and subsequently migrated to northern China. This expansion occurred later than the expansion event of *Bos taurus* in Longdon*g*. A history of plow cultivation and cattle domestication was observed in Longdong, dating to the Xia Dynasty before 2000 BC. During this period, migration and trade among human populations ceased, which hindered the immigration of *Bos indicus* cattle distributed in southern China. Moreover, the evolution of sub-branches of Chinese cattle was detected based on analyses of copy number variations and Y chromosomes [35], and a similar evolution was observed in European cattle based on the mitochondrial genome [20].

## Conclusions

The domestication of cattle has had a long history in northwestern China with the propagation of Zhouzu farming culture. The research based on mtDNA revealed that the native cattle of northwestern China were domesticated from two different maternal ancestors (*Bos taurus* and *Bos indicus*), which migrated to the central plains of China from the north and south, respectively, with *Bos taurus* remaining at the edges of the region. The population expansion of the cattle domesticated from *Bos taurus* occurred in Longdong of Gansu Province, and these cattle formed four relatively independent evolutionary branches. Subsequent to this expansion event, *Bos indicus* migrated from southern to northern China.

## Supporting information

S1 Fig Geographic locations of the samples considered in the current study. The numbers refer to the sample IDs in S1 Table. *Bos taurus taurus* migrated from the north, and *Bos taurus indices* migrated from the south and kept moving after intersecting in the central plains of China. Thus, individuals of Clade T (Ta, Tb, Tc and Td) were more common in Xingjiang (89.74%), Hexi of Gansu (100%), Inner Mongolia (94.44%), Ningxia (78.67%), Qinghai (76.74%) and the Longdong region of Gansu (78.67%) than in Shanxi (68.36%) and Sichuan (69.07%), but the opposite results were observed for Clade I (Table 1).
(TIF)

S1 Table Sample information. (DOC)

S2 Table Sequence variations of 258 mtDNA haplotypes in 698 domestic cattle (*Bos taurus taurus*). The numbers of individuals sharing a haplotype are listed in the right column. The sequence of *Bos taurus taurus* (V00654, Anderson et al., 1982) was used as a reference sequence. The fragments sequenced in the current study covered positions 1 to 410 relative to the reference sequence. Dots (•) and hyphens (–) denote identity with the reference sequence and missing data, respectively.
(DOC)

S3 Table The key to haplotype name is based on the variations in mtDNA D-loop. Reference: Cattle and aurochs mtDNA tree Build 1 (Jun 1, 2014) https://www.dometree.org/trees/cattle.htm); Nucleotide position numbers are relative to the V00654 (BRS). The control region mutations: 15903–16313; The haplotype names were showed in brackets after mutations.
(XLS)
