# Supplementary figures and images for: Maternal genetic and phylogenetic characteristics of domesticated cattle in northwestern China
Source: PLoS One. 2018 Dec 27;13(12):e0209645. doi: 10.1371/journal.pone.0209645 (PMC6307701; doi:10.1371/journal.pone.0209645)

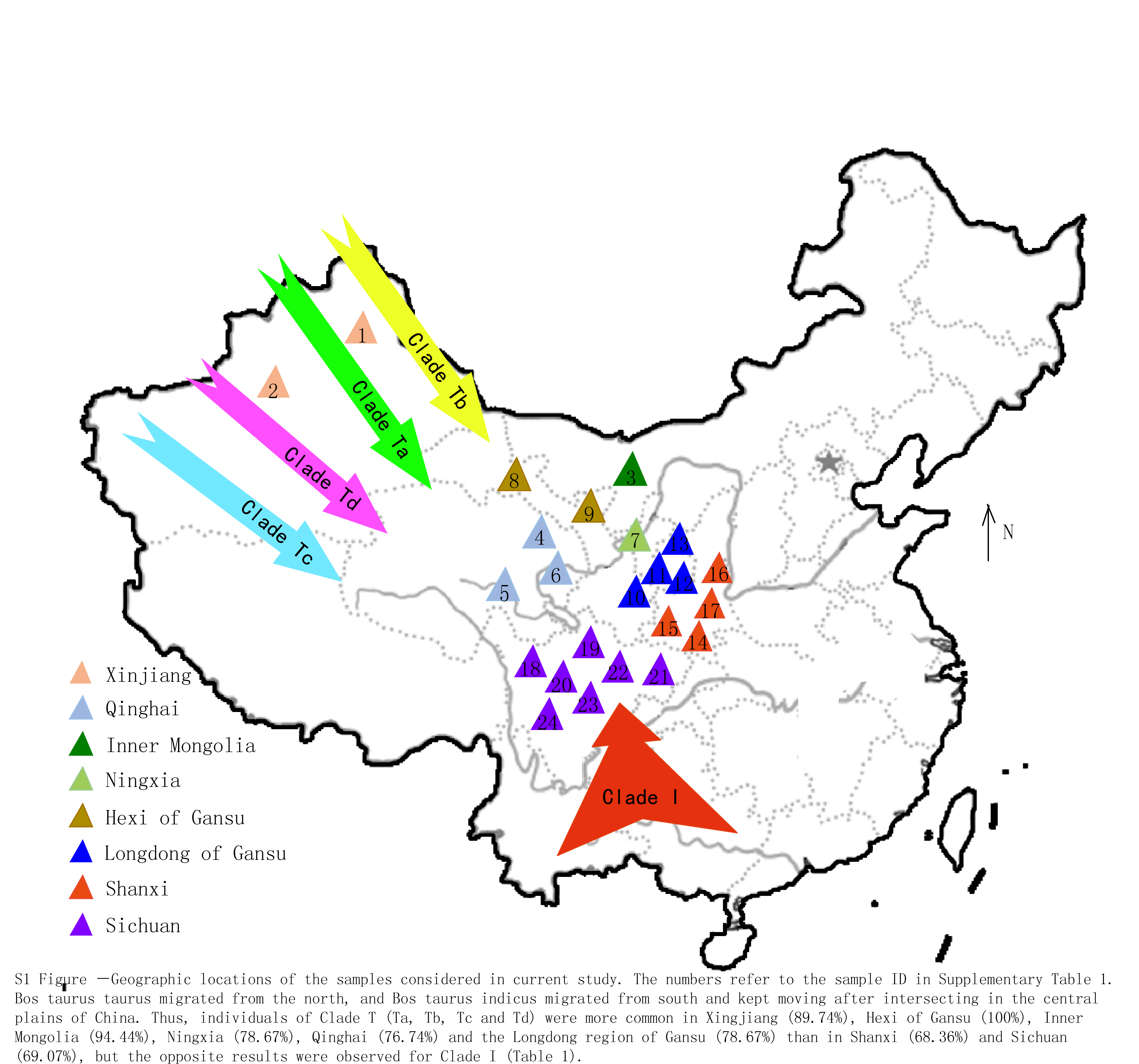

Supplement: S1 Fig — The numbers refer to the sample IDs in S1 Table. Bos taurus taurus migrated from the north, and Bos taurus indices migrated from the south and kept moving after intersecting in the central plains of China. Thus, individuals of Clade T (Ta, Tb, Tc and Td) were more common in Xingjiang (89.74%), Hexi of Gansu (100%), Inner Mongolia (94.44%), Ningxia (78.67%), Qinghai (76.74%) and the Longdong region of Gansu (78.67%) than in Shanxi (68.36%) and Sichuan (69.07%), but the opposite results were observed for Clade I (Table 1). (TIF) [file pone.0209645.s001.tif]
