# Supplementary material for: Maternal genetic and phylogenetic characteristics of domesticated cattle in northwestern China
Source: PLoS One. 2018 Dec 27;13(12):e0209645. doi: 10.1371/journal.pone.0209645 (PMC6307701; doi:10.1371/journal.pone.0209645)
Supplement: S1 Table — (DOC) [file pone.0209645.s002.doc]

# S1 Table Sample information

| **Sample ID** | **Breeds / Subspecies** | **Haplotypes** ^a^ | **No.** | **Location** | **Accession No. and Sources** |
| --- | --- | --- | --- | --- | --- |
| 1 | Aletai (XAT) | TA1(1), TA2(3), TA34(1), TA48(5), TB1(2), I2(2) | 14 | Aletai, Xinjiang | EU281359-72: (Jia et al., 2010) |
| 2 | Kazakh (XKS) | TA1(3), TC2(1), TD1(1) | 5 | Tacheng, Xinjiang | EU281432: (Jia et al., 2010);  DQ166049-52 ^c^ |
| 3 | Anxi (GAX) | TA1(1), TA77(4), TB2(1), TC19(1), TC31(1), TD1(1) | 9 | Anxi, Hexi of Gansu | AY521076-82, AY902382-83: (Lai et al., 2005) |
| 4 | Wuwei Yellow (GWW) | TA12(1), TB31(1), TB34(1),TC3(1), TC22(1), TC45(1) | 6 | Wuwei, Hexi of Gansu | AY378127-32 ^d^ |
| 5 | Mongolia (MMG) | TA1(1), TA8(1), TA40(2), TA70(2), TA81(2), TA87(1), TB2(1), TB4(1), TB34(2), TC34(4), I14(1) | 18 | Inner Mongolia | DQ166053-57 ^c^; EU281447-54: (Jia et al., 2010); DQ344951-52: (Lei et al., 2006); AY115529, AY119675,79 ^b^ |
| 6 | Guyuan (NGY) | TA1(6), TA3(1), TA5(1), TA8(1), TA11(1), TA20(2), TA22(1), TA40(1), TA44(1), TA51(1), TA53(1), TA66(1), TA74(1), TA77(1), TA79(1), TA87(4), TA89(1), TA91(1), TA98(2), TA99(1), TA100(1), TB6(1), TB4(2), TB13(1), TB27(1), TB35(1), TC2(2), TC6(1), TC16(1), TC23(1), TC35(1), TC38(1), TC51(1), TD1(4), TD2(1), TD4(1), TD5(1), TD11(1), TD18(1), TD22(1), TD27(1), TD30(1), TD31(1), TD32(1), I1(9), I16(2), I17(1), I28(2), I30(2) | 75 | Gucheng village, Pengyang county Guyuan city, Ningxia Hui Autonomous Region  (106°46′57″N, 35°86′28″E) | This study |
| 7 | Qinghai Yellow (QQY) | TA40(1) | 1 | Xunhua, Qinghai | AB177765: (Shi et al., 2008) |
| 8 | Haixi (QX) | TA1(6), TA8(1), TA30(1), TA50(1), TA57(1), TA60(1), TA68(1), TA75(1), TA84(1), TA94(1), TA101(1), TB4(1), TB33(1), TC2(4), TC40(1), TC42(1), TC49(1), TD1(2), TD8(1), TD9(1), TD22(1), I1(1), I11(1), 116(1), I24(1), I27(2) | 36 | Hedong village, Wulan county, Haixi city, Qinghai province, (98°52′20″N, 36°92′36″E) | This study |
| 9 | Haidong(QD) | TA1(8), TA10(1), TA28(1), TA54(1), TA61(1), TA77(2), TA90(1), TA95(1), TA98(1), TA105(1), TB4(2), TB5(1), TB10(1), TB13(1), TB14(1), TB17(1), TB19(1), TB28(1), TC2(4), TC7(1), TC10(1), TC11(2), TC15(1), TC30(2), TD1(5), TD18(1), TD29(1), I1(1), I10(1), I11(1), I34(1) | 49 | Shangtan village, Ledu county, Haidong city, Qinghai province,  (102°18′09″N, 36°38′86″E) | This study |
| 10 | Jingchuan (GZJ) | TA1(4), TA10(1), TA21(1), TA35(2), TA40(1), TA45(1), TA60(1), TA69(1), TA70(1), TA71(1), TA72(1), TA77(1), TA85(2), TA88(1), TA96(1), TB4(2), TB7(1), TB8(1), TB16(1), TB18(1), TB20(1), TB21(1), TB22(1), TB24(1), TB25(1), TB29(1), TB32(1), TB33(1), TC1(1), TC2(4), TC9(1), TC12(1), TC20(1), TD1(3), TD13(1), TD16(1), TD29(1), I1(9), I2(2), I16(1), I26(1), I28(1), I32(1) | 63 | Xuwang village, Jingchuan county, Pingliang city, Gansu province, (107°18′79″N, 35°43′36″E) | This study |
| 11 | Qingcheng  (GZQ) | TA1(1), TA8(2), TA17(1), TA31(1), TA33(1), TA39(1), TA49(2), TA57(2), TA67(1), TA77(1), TA86(1), TA87(1), TA101(1), TB4(1), TB13(2), TC2(4), TC3(1), TC1792), TD1(3), TD5(1), TD7(1), TD22(1), TD24(2), I1(7), I7(1), I12(1), I22(1) | 44 | Jiaqiao village, Qingcheng county, Qinagyang city, Gansu province, (107°90′53″N, 36°08′30″E) | This study |
| 12 | Ningxian (GZN) | TA1(3), TA9(1), TA15(1), TA26(1), TA30(1), TA38(1), TA39(1), TA52(1), TA54(1), TA56(1), TA63(1), TA67(1), TA77(2), TA78(1), TA80(1), TA87(1), TA93(1), TA102(1), TB2(1), TB4(2), TB9(1), TB15(1), TB28(1), TC2(3), TC5(1), TC14(1), TC19(1), TC28(1), TC29(1), TC30(1), TC44(1), TD1(3), TD5(1), TD6(1), TD23(1), TD24(1), TD25(1), TD27(1), I1(7), I4(1), I5(1), I15(1), I16(1), I22(1) | 58 | Beijie village, Ning county, Qinagyang city, Gansu province, (108°02′30″N, 35°41′53″E) | DQ166063-66 ^c^; This study |
| 13 | Zhenyuan (GZZ) | TA1(3), TA8(1), TA14(1), TA16(1), TA20(2), TA37(1), TA54(1), TA57(1), TA60(1), TA64(1), TA77(2), TA81(1), TA98(3), TA104(1), TA106(1), TB4(1), TB14(1), TB25(2), TB26(2), TB30(1), TB35(1), TC2(4), TC33(1), TD1(1), TD5(1), TD24(1), TD28(1), I1(6), I16(1), I31(1) | 46 | Zhaojiagouquan village, Zhengning county, Qinagyang city, Gansu province, (108°33′76″N, 35°50′59″E) | This study |
| 14 | Hanzhong (SQH) | TA1(2), TA14(1), TA27(1), TA31(1), TA46(1), TA67(1), TA87(2), TB4(1), TB11(1), TB20(1), TC2(1), TC36(1), TD1(3), I1(2) | 19 | Hanzhong, Shanxi | DQ166083-89 ^c^; AF514784 ^e^; Y521107-11, AY902395: (Lai et al., 2005); DQ344959-61, AY119673-74 ^b^ |
| 15 | Baoji (SQB) | TA1(3), TA4(1), TA6(1), TA8(1), TA19(1), TA20(2), TA23(1), TA47(1), TA52(1), TA55(1), TA73(1), TA77(1), TA83(1), TA87(1), TA97(1), TA101(1), TA106(1), TB4(1), TB9(1), TB23(1), TC2(3), TC4(1), TC8(1), TC10(1), TC24(1), TC48(1), TD1(2), TD13(1), TD23(1), TD30(1), I1(8), I3(1), I9(1), I13(1), I16(1), I18(1), I19(2), I23(1), I24(1), I25(1) | 54 | Yangjia Village, Mei county, Baoji city, Shanxi province, (107°82′91″N, 34°30′81″E) | This study |
| 16 | Yanan (SQY) | TA1(3), TA10(1), TA18(1), TA39(1), TA42(1), TA62(1), TA66(1), TA76(1), TA87(2), TA92(1), TB25(1), TC13(1), TC2(7), TC21(1),TC25(1), TC32(1), TC50(1), TD1(2), TD15(1), TD16(1), TD30(1), I1(11), I2(1), I16(3), I17(1), I20(1), I21(1) | 49 | Caodian village, Huanglong county, Yanan city, Shanxi province, (109°86′00″N, 35°59′18″E) | This study |
| 17 | Tongchuan (SQT) | TA1(3), TA7(1), TA14(2), TA25(1), TA32(1), TA41(1), TA43(1), TA58(1), TA59(1), TA65(1), TA77(2), TA82(1), TA87(1), TA103(1), TB3(1), TB15(1), TB16(1), TB20(1), TB26(1), TB28(1), TC2(4), TC11(1), TC13(2), TC46(1), TD1(3), TD12(1), TD30(1), I1(14), I8(1), I16(3) | 55 | Caoyuan village, Yijun county, Tongchuan city, Shanxi province, (109°21′81″N, 35°45′54″E) | This study |
| 18 | Sanjiang (SSJ) | TA36(1), TC2(1), TC17(2), TD1(1), I1(2) | 7 | Wenxian, Sichuan | AY521112-16, AY902396-97: (Lai et al., 2005) |
| 19 | TongJiang (STJ) | TA8(1), TA24(1), TA47(10), TA58(1), TB4(3), TC2(4), TC11(1), TC18(1), TC22(1), TC26(2), TC39(3), TC41(1), TC47(2), TC48(1), TD1(2), TD19(2), TD20(1), I1(14), I2(2), I11(1) | 54 | Tongjiang, Sichuan | EF417933-86: (Chen et al., 2008) |
| 20 | Xizhen (SXZ) | TA98(1), TC2(2), TC13(1), TD29(2), I1(1),I24(2), I29(1) | 10 | Xizhen, Sichuan | DQ344969-70: (Lei et al., 2006);  DQ166096-100 ^c^; AY119670-71, 77 ^b^ |
| 21 | Xuanhan (SXH) | TA18(1), TA29(1), TC13(1), TC27(1), TC52(1), TD1(1), I1(2), I29(1), I33(1) | 10 | Xuanhan, Sichuan | DQ166107-113 ^c^; DQ344962-64: (Lei et al., 2006) |
| 22 | Hanyuan (SHY) | TA13(1), TA77(1), TA87(1), TC37(1) | 4 | Hanyuan, Sichuan | AY521090-93: (Lai et al., 2005) |
| 23 | Bashan (SBS) | TB12(1), TC2(1), TD1(2), I1(2), I23(1) | 7 | Bashan, Sichuan | AY521083-87, AY902385-86: (Lai et al., 2005) |
| 24 | Ebian (SEB) | TC2(3), TC48(2) | 5 | Ebian, Sichuan | AY521088-89, AY902387-89: (Lai et al., 2005) |

^a^ The number of individuals shared the same haplotype in each breed was in parentheses.

^b^ Data was published only in GenBank by Lei et al. in 2002.

^c^ Data was published only in GenBank by Cai et al. in 2005.

^d^ Data was published only in GenBank by Qi et al. in 2003.

^e^ Data was published only in GenBank by Chen et al. in 2002.
