# Supplementary material for: Maternal genetic and phylogenetic characteristics of domesticated cattle in northwestern China
Source: PLoS One. 2018 Dec 27;13(12):e0209645. doi: 10.1371/journal.pone.0209645 (PMC6307701; doi:10.1371/journal.pone.0209645)
Supplement: S2 Table — The numbers of individuals sharing a haplotype are listed in the right column. The sequence of Bos taurus taurus (V00654, Anderson et al., 1982) was used as a reference sequence. The fragments sequenced in the current study covered positions 1 to 410 relative to the reference sequence. Dots (•) and hyphens (–) denote identity with the reference sequence and missing data, respectively. (DOC) [file pone.0209645.s003.doc]

# S2 Table. Sequence variations of 258 mtDNA haplotypes in 698 domestic cattles (*Bos taurus taurus*)

| **Haplotype** | 16812334455556888891111111111111111111111111111111111111111122222222222222222222222222222222222222333333333333333333333333333333444  0071901792035620011123444444455555555666777777888888999900001111111222222333333444566678899999002222344444555667778899999999001  2634708045678901234569056012357012345012906780134579023458135679347101242634578165689145678257161450501236789080 | **Frequency** |
| --- | --- | --- |
| V00654 | CGTGCGGGTACGGATTAAATAATTGATTGACCTCTATAGCTAATTATATAGACTGTAGTTGTTCTTTTGTGTTATCTTTTTCTATGAATTTGCGGCGGTCAACCTCCAATTTCGTCTCGAATCTAACGGTT |  |
| TA1 | ................................................................................................................................... | 48 |
| TA2 | .....................................................C..........................C.................................................. | 3 |
| TA3 | .....................................................C..........................C....A............................................. | 1 |
| TA4 | .....................................................C...............................A............................................. | 1 |
| TA5 | ..............................................C......................................A......................G...................... | 1 |
| TA6 | ....................................................T.............................................................................. | 1 |
| TA7 | ....................................................T................................A............................................. | 1 |
| TA8 | ....................................C.............................................................................................. | 8 |
| TA9 | ....................................C...........................................................A.................................. | 1 |
| TA10 | ....................................C.........................................................................................T.... | 3 |
| TA11 | ................................C.....A.C........................C................................................................. | 1 |
| TA12 | ................................C.......C.......................................................................................... | 1 |
| TA13 | ................................C.......C...................................................A.................................T.... | 1 |
| TA14 | ................................C.......C.....................................................................................T.... | 4 |
| TA15 | ................................CT..C.............................................................................................. | 1 |
| TA16 | ..........................C.........C...........................................C.....G............................................ | 1 |
| TA17 | .........................................G......................................C.................................................. | 1 |
| TA18 | ..C..............G..................C...........................C.................................................................. | 2 |
| TA19 | ..C.................................C...........................C......................T........................................... | 1 |
| TA20 | ..C.................................C...........................C.................................................................. | 6 |
| TA21 | ..C.....................A...........C...........................C.............................A.................................... | 1 |
| TA22 | ..C.................................C...........................C..............................................................A... | 1 |
| TA23 | ..C.............................................................C....................A............................................. | 1 |
| TA24 | ..C.............................................................C.................................................................. | 1 |
| TA25 | ......................................C.......................CT......A....................A..................C.................... | 1 |
| TA26 | ......................................C.......................C.......A....................A..............T...C.................... | 1 |
| TA27 | ......................................C...............................A....................A..............T.....TA................. | 1 |
| TA28 | ......................................C....................................................A....................................... | 1 |
| TA29 | ..........G.......................................................................C...........................C.................... | 1 |
| TA30 | ..........G...................................................................................................C.................... | 2 |
| TA31 | ..............................................................................................................C.................... | 2 |
| TA32 | ......................C.......................................................................................C.................... | 1 |
| TA33 | ......................C............................................................................................................ | 1 |
| TA34 | T.....................................AT........................................................................................... | 1 |
| TA35 | .......................................T........................................................................................... | 1 |
| TA36 | ....................................C..T........................................................................................... | 1 |
| TA37 | .A..............................................................................................................TA................. | 1 |
| TA38 | .A................................................................................................................................. | 1 |
| TA39 | ...........................................................................T....................................................... | 3 |
| TA40 | ....................................C.................................................................T............................ | 5 |
| TA41 | ..............................T.......................................................................T............................ | 1 |
| TA42 | ......................................................................................................T............................ | 1 |
| TA43 | ......................................................................................................T...T........................ | 1 |
| TA44 | .....................G..A............................................C..........CT....................T............................ | 1 |
| TA45 | ........................A.....................C..................................T....................T............................ | 1 |
| TA46 | ..................................................................................................C................................ | 1 |
| TA47 | ..................................C................................................................................................ | 11 |
| TA48 | ...................................................G............................................................................... | 5 |
| TA49 | .......................................................................................................T........................... | 2 |
| TA50 | ....................................C................................................................G.T........................... | 1 |
| TA51 | .................................................................................T................................................. | 1 |
| TA52 | ......................................................A............................................................................ | 2 |
| TA53 | ......................................................A..................G......................................................... | 1 |
| TA54 | ......................................................A..........................T................................................. | 3 |
| TA55 | ..............................................................................................A.................................... | 1 |
| TA56 | .............................................................C..................................................................... | 1 |
| TA57 | .............................................................C............................................T........................ | 4 |
| TA58 | ...............................T..............................................................................C.................... | 2 |
| TA59 | ...............................T........................G...............C.....................................C.................... | 1 |
| TA60 | ...............................T..............................C...............................................C.................... | 3 |
| TA61 | ...............................T..............................C..................T............................C.................... | 1 |
| TA62 | ........................A......T.....G.....C.....................C............................................C.................... | 1 |
| TA63 | ...............................T.................................C............................................C.................... | 1 |
| TA64 | ...............................T..........G...C..........................................................T....C.................... | 1 |
| TA65 | ...............................T................................................................................................... | 1 |
| TA66 | ........................A.......................................................................................................... | 2 |
| TA67 | ........................A.......................................................................A.................................. | 3 |
| TA68 | ........................A........................................C......CG......................................................... | 1 |
| TA69 | ....................................................................................................................A.............. | 1 |
| TA70 | ....T.............................................................................................................................. | 3 |
| TA71 | .........................................................................................C......................................... | 1 |
| TA72 | .....A............................................................................................................................. | 1 |
| TA73 | ..................................................................................................................G................ | 1 |
| TA74 | ...........................................................................................................G....................... | 1 |
| TA75 | .............................................G..................................................................................... | 1 |
| TA76 | .............................................................................................................................G..... | 1 |
| TA77 | ..............................................................................................................................T.... | 17 |
| TA78 | ......................................................................A.......................................................T.... | 1 |
| TA79 | ....................................................................A............T............................................T.... | 1 |
| TA80 | ....................................................................A.........................................................T...C | 1 |
| TA81 | ......................................A.............................A.........................................................T.... | 3 |
| TA82 | ....................G..............T...........................T..............................................................T.... | 1 |
| TA83 | ...................C...........................................T................................................................... | 1 |
| TA84 | ............C.............................................................................................T........................ | 1 |
| TA85 | ..........................................................................................................T......................C. | 2 |
| TA86 | ............................................C.............................................................T........................ | 1 |
| TA87 | ..........................................................................................................T........................ | 14 |
| TA88 | ..........................................................................................................T..............C......... | 1 |
| TA89 | .........G....................................................................C...........................T...................T.... | 1 |
| TA90 | ...............................................................................C..........................T........................ | 1 |
| TA91 | ......................................................................AC........................................................... | 1 |
| TA92 | ..............................T........................................C........................................................... | 1 |
| TA93 | .......................................................................C..................................T........................ | 1 |
| TA94 | .......................................................................C........................A.............................T.... | 1 |
| TA95 | .......................................................................C..C...................................................T.... | 1 |
| TA96 | ........................................................................................C.......................T.................. | 1 |
| TA97 | ........................................................................................C.......................................... | 1 |
| TA98 | .................................................................C................................................................. | 7 |
| TA99 | .................................................................C.....C........................................................... | 1 |
| TA100 | ..................................................................................C......................T........................C | 1 |
| TA101 | .........................................................................................................T......................... | 3 |
| TA102 | .................................................................C.......................................T.T.........T............. | 1 |
| TA103 | ......................................A................................C.............A...................T......................... | 1 |
| TA104 | ..........G..............................................................................................T....C..A.......C......... | 1 |
| TA105 | ........C.G.......G...........T.C......T......C......C.................C..............................T..T....C..A................. | 1 |
| TA106 | ........C.G.......G.........A.T.C......T......C......C.................C..............................T..T....C..A................. | 2 |
| TB1 | ......................................C....................................................A....A.............C.................... | 2 |
| TB2 | ......................................C........................G...........................A..................C.................... | 3 |
| TB3 | ......................................C........................G.....................A.....A..................C.................... | 1 |
| TB4 | ......................................C....................................................A..................C.................... | 17 |
| TB5 | ..........T...........................C....................................................A..................C.................... | 1 |
| TB6 | .............G........................C....................................................A..................C.................... | 1 |
| TB7 | .............G...............G........C.........C..........................................A..................C.................... | 1 |
| TB8 | .............G........................C..............C.....................................A..................C..A................. | 1 |
| TB9 | ......................................C......................C.............................A..................C.................... | 2 |
| TB10 | ......................................C....................................T.....T.........A..................C.................... | 1 |
| TB11 | ......................................C..........................................T.........A..................C.................... | 1 |
| TB12 | ......................................C....................................................A..........T.......C.................... | 1 |
| TB13 | ..............................T.......C....................................................A..................C..............G..... | 4 |
| TB14 | ..............................T.......C.......................C............................A..................C..............G..... | 2 |
| TB15 | ..............................T.......C....................................................A.............T....C.................... | 2 |
| TB16 | ......................................C....................................................A.......T..........C.................... | 2 |
| TB17 | ......................................C....................................................A.............T....C.................... | 1 |
| TB18 | ........................A.............C....C...............................................A..................C.................... | 1 |
| TB19 | ......................................C..........................C.........................A..................C.................... | 1 |
| TB20 | ......................................C....................................................A..............A...C.................... | 3 |
| TB21 | ........C.............C...............C....................................................A..................C.................... | 1 |
| TB22 | ...............C......................C.......C............C...............................A..................C.................... | 1 |
| TB23 | ...............C.............C........C.......C............C.........C.....................A..................C.................... | 1 |
| TB24 | ...............C......................C.......C........................C...................A..................C.................... | 1 |
| TB25 | ......................................C.......................................C............A..................C.................... | 4 |
| TB26 | ......................................C.......................................CC...........A..................C.................... | 3 |
| TB27 | ......................................C.......................................C......A.....A..................C.................... | 1 |
| TB28 | ......................................CT...................................................A..................C...............T.... | 3 |
| TB29 | ......................................C....................................................A..................C...............T.... | 1 |
| TB30 | ...............................T......C....................................................A..................C...............T.... | 1 |
| TB31 | ......................................C............G.................C.....................A..................C...............T.... | 1 |
| TB32 | ......................................C..............................C.....................A..................C.................... | 1 |
| TB33 | ......................................C..............C.....................................A..................C.T.................. | 2 |
| TB34 | ......................................C...............................A....................A..................C.................... | 3 |
| TB35 | ......................................C.......................................C............A....................................... | 2 |
| TC1 | .......................................T.............................C............................................................. | 1 |
| TC2 | .....................................................................C............................................................. | 52 |
| TC3 | .....................................................................C.........C................................................... | 2 |
| TC4 | ............................................................A........C.C........................................................... | 1 |
| TC5 | .....................................................................C.C.................................................C......... | 1 |
| TC6 | .................................................................C...C............................................................. | 1 |
| TC7 | ...........................C.........................................C............................................................. | 1 |
| TC8 | ...........................C..........................A..............C............................................................. | 1 |
| TC9 | ..............C...........................................C.......C..C............................................................. | 1 |
| TC10 | ......................................A..............................C............................................................. | 2 |
| TC11 | ......A..............................................................C............................................................. | 4 |
| TC12 | .....................................................C...............C............................................................. | 1 |
| TC13 | .....................................................................C...................................T......................... | 5 |
| TC14 | T....................................................................C...................................T......................... | 1 |
| TC15 | ...............................................C.....................C............................................................. | 1 |
| TC16 | .................................................G...................C............................................................. | 1 |
| TC17 | .......C.............................................................C............................................................. | 4 |
| TC18 | .....................................................................C..............................G.............................. | 1 |
| TC19 | ..........................................G..........................C............................................................. | 2 |
| TC20 | ..........................................G..........................C.......C..................................................... | 1 |
| TC21 | ..........................................G..........................C...............A..................G.......................... | 1 |
| TC22 | .....................................................................C........................................................T.... | 2 |
| TC23 | .....................................................................C..............C.........................................T.... | 1 |
| TC24 | .....................................................................C.......C................................................T.... | 1 |
| TC25 | .....................................................................C...............A........................................T.... | 1 |
| TC26 | ..........................................G..........................C......................................G..................A... | 2 |
| TC27 | .....................................................................C......................................G...................... | 1 |
| TC28 | .....................................................................C....................G....T...............A...T..AGT.T.C..AT.. | 1 |
| TC29 | .....................................................................C.........................................................A... | 1 |
| TC30 | .....................G...............................................C............................................................. | 3 |
| TC31 | ..............................T......................................C............................................................. | 1 |
| TC32 | .....................................................................C..........................................T.................. | 1 |
| TC33 | .A...................................................................C............................................................. | 1 |
| TC34 | .....................................................................C...........................................A................. | 4 |
| TC35 | ..............................................C......................C............................................................. | 1 |
| TC36 | ..............................................C......................C........................................C.................... | 1 |
| TC37 | .....................................................................C........................................C.................... | 1 |
| TC38 | .....................................................................C.....T....................................................... | 1 |
| TC39 | ....................................................T..........A.....C.....T....................................................... | 3 |
| TC40 | ....................................................T................C.....T....................................................... | 1 |
| TC41 | ....................................................T................C............................................................. | 1 |
| TC42 | .....................................................................CA.................C.......................................... | 1 |
| TC43 | .....................................................................C..................C.............T............................ | 1 |
| TC44 | ........................A............................................C................................T..........A................. | 1 |
| TC45 | .....................................................................C................................T.......C.................... | 1 |
| TC46 | .....................................................................C............................C................................ | 1 |
| TC47 | ...................................................G.................C............................................................. | 2 |
| TC48 | .....................................................................C.................................T........................... | 4 |
| TC49 | ....................................C................................C........................A.................................... | 1 |
| TC50 | ..........................C..................................C..................................................................... | 1 |
| TC51 | .............................................................C.......C............................................................. | 1 |
| TC52 | ...............................T.....................................C............................................................. | 1 |
| TD1 | ..........................C..............................A.....................................................................A... | 39 |
| TD2 | ..........................C..............................A............................G........................................A... | 1 |
| TD3 | ..........................C..............................A...................C.................................................A... | 1 |
| TD4 | ..........................C..............................A.....T...............................................................A... | 1 |
| TD5 | ..........................C..............................A.........................G...........................................A... | 3 |
| TD6 | .A........................C..............................A.........................G...........................................A... | 1 |
| TD7 | ..........................C...T..........................A.....................................................................A... | 1 |
| TD8 | ..........................C..............................A.......................T.............................................A... | 1 |
| TD9 | ..........................C...........A..................A.....................................................................A... | 1 |
| TD10 | ..........................C..............................A...........................A.........................................A... | 1 |
| TD11 | ..........................C..............................A...........................A.................T.......................A... | 1 |
| TD12 | ..........................C..............................A.......C...................A.........................................A... | 1 |
| TD13 | ..........................C..............................A........................C............................................A... | 1 |
| TD14 | ..........................C..............................A........................C..A..........................T..............A... | 1 |
| TD15 | ..........................C..............................A.............C.......................................................A... | 1 |
| TD16 | ..........................C..............................A.............C..............................T........................A... | 1 |
| TD17 | ...A......................C..............................A.....................................................................A... | 1 |
| TD18 | ...A......................C..............................A.............C.......................................................A... | 1 |
| TD19 | ................G.........C....T.........................A................................................T....................A... | 2 |
| TD20 | ..........................C....T.......T.............C...A................................................T....................A... | 1 |
| TD21 | ..........................C..............................A...........C.........................................................A... | 1 |
| TD22 | ..........................C..............................A....C.......................................T........................A... | 3 |
| TD23 | .........................................................A....C.......................................T........................A... | 1 |
| TD24 | ..........................C..............................A............................................T..T.....................A... | 4 |
| TD25 | ..........................C..............................A............................................T........................A... | 1 |
| TD26 | ..........................C..................G.........................................................T.......................A... | 1 |
| TD27 | ..........................C....................................................................................................A... | 1 |
| TD28 | .........................................................A.........C...........................................................A... | 1 |
| TD29 | .........................................................A.....................................................................A... | 8 |
| TD30 | .........................................................A........................C.............A..............................A... | 1 |
| TD31 | .......................C.................................A..............................C......................................A... | 1 |
| I1 | ........C.GA......G.....A.....T.......AT......C...A.T.......A.C..C.CACAC....C..CC...C........A.......G...TT..................GT.... | 96 |
| I2 | ........C.GA......G.....A.............AT......C...A.T.......A.C..C.CACAC....C..CC...C........A.......G...TT..................GT.... | 7 |
| I3 | ........C.GA......G.....A.....T.......AT......C...A.TC......A.C..C.CACAC....C..CC...C........A.......G...TT..................GT.... | 1 |
| I4 | ........C.GA......G.....A.....T.......AT......C...A.T.......A.C..C.CACAC....C..CC...C........A.......G...TT................A.GT.... | 1 |
| I5 | ........C.GA......G.....A.....T.......AT......C...A.T..G....A.C..C.CACAC....C..CC...C........A.......G...TT..................GT.... | 1 |
| I6 | ........C.GA......G.....A...A.T.......AT......C...A.T.......A.C..C.CACAC....C..CC...C........A.......G...TT..................GT.... | 1 |
| I7 | ........C.GA......G.....A.....T.......AT......C...A.T.......A.C..C.CACAC....C..CC...C........A.......G..GTT..G....G..........GT.... | 1 |
| I8 | ........C.GA......G.....A.....T.......AT......C...A.T.......A.C..C.CACAC..C.C..CC...C........A.......G...TT..................GT.... | 1 |
| I9 | ........C.GA......G.....AG...GT.......AT......C...A.T.......A.C..C.CACAC....C..CC...C........A.......G...TT..................GT.... | 1 |
| I10 | ........C.GA...C..G.....A.....T.......AT......C...A.T.......A.C..C.CACAC....C..CC...C........A.......G...TT..................GT.... | 1 |
| I11 | ........C.GA......G.....A.....T.......AT......C...A.T.......A.C..C.CACAC....C..CC...C........A....C..G...TT..................GT.... | 3 |
| I12 | .A......C.GA......G.....A.....T.......AT......C...A.T.......A.C..C.CACAC....C..CC...C........A.......G...TT..................GT.... | 1 |
| I13 | ........C.GA......G..G..A.....T.......AT......C...A.T.......A.C..C.CACAC....C..CC...C........A.......G...TT..................GT.... | 1 |
| I14 | ........C.GA......G.....A.....T.......AT......C...A.T.......A.C..C.CACAC....C..CC...C........A.......G...TT......A...........GT.... | 1 |
| I15 | ........C.GA......G.....A.....T.......AT......C...A.T.......A.C..C.CACAC....C..CC...C.T......A.......T...TT.T................GT.... | 1 |
| I16 | ........C.GA......G.....A.....T.......AT......C...A.T.......A.C..C.CACAC....C..CC...CA.......A.......G...TT..................GT.... | 12 |
| I17 | ........C.GA......G.....A.....T.......AT......C...A.T.......A.C..C.CACAC....C..CC...CA.......A..A....G...TT..................GT.... | 2 |
| I18 | ........C.GA......G.....A.....T.......AT......C...A.T.......A.C..C.CACAC....C..CC...CA.......A..A....G.T.TT..................GT.... | 1 |
| I19 | ........C.GA......G.....A.....T.......AT......C...A.T.......A.C..C.CACAC....C..CC...C.G......A.......G...TT..................GT.... | 2 |
| I20 | ........C.GA......G.....A.....T.......AT......C...A.T.......A.C..C.CACAC....C..CC...CAG......A.......G...TT..................GT.... | 1 |
| I21 | ........C.G.......G.....A.....T.......AT......C...A.T.......A.C..C.CACAC....C..CC...CA.......A.......G...TT..................GT.... | 1 |
| I22 | ........C.GA......G.....A.....T.......AT..G...C...A.T.......A.C..C.CACAC....C..CC...C........A.......G...TT..................GT.... | 2 |
| I23 | ........C.GA......G.....A.....T.......AT..........A.T.......A.C..C.CACAC....C..CC...C........A.......G...TT..................GT.... | 2 |
| I24 | ........C.GA......G.....A.....T...C...AT..........A.T.......A.C..C.CACAC....C..CC...C........A.......G.T.TT..................GT.... | 4 |
| I25 | ........C.GA......G.....A.....TT......AT..........A.T.......A.C..C.CACAC....C..CC...C........A.......G...TT..................GT.... | 1 |
| I26 | ........C.GA......G.....A.....TT......AT......C...A.T.......A.C..C.CACAC....C..CC...C........A.......G...TT..................GT.... | 1 |
| I27 | ........C.GA......G.....A.....T.......AT......C...A.T.......A.C..C.CACAC....C..CC...C........A.......G...TT..................G..... | 2 |
| I28 | ........C.GA......G.....A.....T.......AT......C...A.T.......A.C..C.CACAC....C..CC...C........A...........TT..................GT.... | 3 |
| I29 | ........C.G.......G.....A.....T.......AT......C...A.T.......A.C..C.CACAC....C..CC...C........A.......G...TT..................GT.... | 2 |
| I30 | ........C.GA......G...........T.......AT......C...A.T.......A.C..C.CACAC....C..CC...C........A.......G...TT..................GT.... | 2 |
| I31 | ........C.GA......G...........T.......AT......C...A.T.......A.C..C.CACAC....C..CC...C........A.......G...TT..................GTA... | 1 |
| I32 | ........C.GA......G.....A.....T.......AT......C...A.T.......A.C..C.CACAC....C..CC...C........A.......G...TT...................T...C | 1 |
| I33 | ........C.GA......G.....A.....T.......AT......C...A.T.......A.C....CACAC....C..CC............A.......G...TT..................GT.... | 1 |
| I34 | ........C.GA......G...........T.......AT......C.....T.........C..C.C.CAC....C..CC...C........A.......G...TT..................GT.... | 1 |

The numbers of individuals sharing a haplotype were listed in the right column. The sequence of *Bos taurus taurus* (V00654, Anderson et al., 1982) was used as a reference sequence. The fragments sequenced in the current study covered position 1 to 410 relative to the reference sequence. Dots (•) and hyphens (?) denote identity with the reference sequence and missing data, respectively.
